# Phoniatrics

**DOI:** 10.1155/2015/156014

**Published:** 2015-10-20

**Authors:** Haldun Oguz, Markus Hess, Adam M. Klein

**Affiliations:** ^1^Department of Otolaryngology, Faculty of Medicine, Yüksek İhtisas Üniversitesi, Ankara, Turkey; ^2^Department of Voice-Speech-Hearing Disorders, University Medical Center Hamburg-Eppendorf, Hamburg, Germany; ^3^Voice Center, Emory University, Atlanta, GA, USA

Phoniatrics is the medical specialty for communicative disorders. It is related with the normal, pathological, and professional processes that involve voice, speech, language, swallowing, and hearing. Besides being a specific discipline, it is also a discipline that combines the accumulation of knowledge from both medical and nonmedical fields of science. Most important medical specialties that contribute to phoniatrics are otorhinolaryngology, neurology, pediatrics, psychiatry, pediatric psychiatry, dentistry, gastroenterology, geriatrics, endocrinology, radiology, genetics, and physical therapy. Otorhinolaryngology (ORL) Section and Board of European Union of Medical Specialists (UEMS) decided “phoniatrics—communication disorders” as one of the seven subspecialities of otorhinolaryngology in 2010 [1]. Many nonmedical specialties also contribute to phoniatrics. These may be named as speech and language pathology, audiology, linguistics, child development, psychology, pediatric psychology, physiotherapy, acoustics, physics, biomedical sciences, pedagogy, and computer information technologies.

The field of* voice* ranges from acoustics and aerodynamics, to the biomechanics of voice, articulation, and resonance, to voice training, vocal ergonomics, and vocal hygiene, to the complex neurologic, psychiatric, and sociological basis of interpersonal communication. The incidence of voice disorders in the general population is considered between 3% and 9% [2, 3]. This incidence is considered much higher for children, being reported up to 24% [4]. These rates only highlight the importance of increasing awareness of voice disorders.

Understanding vocal production is a crucial step in diagnosing and treating voice ailments. In this issue, you will find an article by A. A. Poznyakovskiy et al. that discusses the importance of vocal tract morphology in voice production by using a 3-tesla magnetic resonance imaging model. They showed that the method is suitable for an improved in-detail analysis of the vocal tract morphology during speech and singing. The development of new diagnostic tools continues to improve our ability to properly identify vocal pathologies. To this end, E. Gonzalez-Moreira and colleagues discussed the utilization of computational speech analysis to identify dementia in elderly patients. Also in this issue, L. Moro-Velázquez and colleagues discuss the efficacy of automatized evaluation of subjective voice parameters by modulation spectra morphological parameters. They demonstrate efficiencies of 81.6% and 84.7% for Grade and Roughness, respectively, using modulation spectra parameters. In another article, L. M. T. Jesus et al. pointed out the acoustic correlates of compensatory adjustment in vocal fold paralysis patients. Proper understanding and diagnosis lead to optimization of vocal restoration. This issue shares several modern methods of managing laryngeal pathology. B. Svensson and colleagues continued to work on restoring vocal fold function after injury in an animal study. In their study they have shown that human embryonic stem cells transplanted to injured rabbit vocal folds restored their vibratory characteristics. H. K. Byeon and colleagues reported their experience with pulsed dye laser in treatment of hemorrhagic vocal fold polyps. It was shown that PDL-assisted enucleation laryngeal microsurgery for the treatment of hemorrhagic vocal polyps can be a safe and effective surgical technique, with improvements in subjective voice perception. 

*Speech and Language Disorders Encompass Articulation Disorders*. Diagnostics, evaluation methods, intelligibility, motor examination, nasalance, preventive counselling, speech therapy, and medical and surgical treatment; developmental language disorders: psychomotor, cognitive, auditory, and language development of normal and diseased children, evaluation of verbal and nonverbal communication, psychomotor, vestibular, and kinesthetic development and disorders, prevention, and rehabilitation; acquired language disorders: evaluation of verbal and nonverbal communication, speech perception, speech and motor evaluation, neurological, laboratory and radiological examination, rehabilitation, and treatment are all discussed. The first human speech dates to at least 50,000 years ago [5]. However, diagnosing and treating speech pathologies are still a clinical challenge. The prevalence of speech sound disorders in children is 8-9%. By the first grade, nearly 5% of children have speech disorders. Most of these disorders are idiopathic in origin [2]. The prevalence of combined receptive and expressive language disorders in children below 7 years ranges between 2 and 3% [6]. The prevalence of language impairment in kindergarten children is 8% [7]. Anyone can acquire aphasia at any age. However, most of the patients fall into an age group that involves middle to late adulthood. 0.3% of U.S. population currently suffer from aphasia [2, 3]. Stuttering is seen in all ages but it is most frequent in young children between the ages of 2 and 6, while they are developing language. Boys are more prone to stuttering during this period. The prevalence of stuttering is estimated to be less than 1% in adults [2]. In the following pages, C. Reuterskiöld and M. I. Grigos are discussing the influence of repeating real words and nonwords on speech motor control in children.

*Swallowing Disorders*. Diagnosis, developmental stages of swallowing, drooling, penetration, retention, regurgitation, aspiration, endoscopic and radiological evaluation, rehabilitation, and medical and surgical treatment are all discussed. Swallowing disorders are seen more frequently in the elderly. The prevalence of solid food dysphagia is 7% in elderly patients [8]. This makes the field of phoniatrics more important in today's ageing society. Swallowing disorders are also more frequent in patients with gastroesophageal reflux and in patients with neurological diseases or with a history of cerebrovascular stroke [9]. Untreated or poorly treated swallowing disorders may result in dehydration, malnutrition, respiratory pathologies, and death [9]. 

*Hearing Disorders*. Physiology and pathology of hearing, tympanometry, audiological evaluation, otoacoustic emission, evoked response audiometry, sensorineural, conductive, and mixed hearing loss, diseases of external, middle, and inner ear, diseases of cochlear nerve, brainstem, and auditory cortex, auditory processing disorders, cochlear implants, hearing aids, prevention, medical and surgical treatment, rehabilitation, and tinnitus are all discussed. According to World Health Organization estimates, 5.3% of world's population have disabling hearing loss [10]. 9% of these are children. Approximately one-third of population above 65 years are affected by disabling hearing loss [10]. The article by Z. Y. T. Chan and B. McPherson discusses the appropriateness of the over-the-counter hearing aids for the demand in the presbycusis. In this issue, you will also find an interesting article by S. S. Grasel and colleagues that discusses the use of auditory steady state responses in evaluation of children with severe hearing loss. Another important paper by M. Coene is discussing the spoken word recognition errors in speech audiometry on normal subjects with complete speech intelligibility.
